# Whistler-mode chorus waves at Mars

**DOI:** 10.1038/s41467-023-38776-z

**Published:** 2023-06-06

**Authors:** Shangchun Teng, Yifan Wu, Yuki Harada, Jacob Bortnik, Fulvio Zonca, Liu Chen, Xin Tao

**Affiliations:** 1 Deep Space Exploration Laboratory/School of Earth and Space Sciences, University of Science and Technology of China, Hefei, China; 2 CAS Center for Excellence in Comparative Planetology/CAS Key Laboratory of Geospace Environment, University of Science and Technology of China, Hefei, China; 3 State Key Laboratory of Marine Geology, School of Ocean and Earth Science, Tongji University, Shanghai, China; 4 Department of Geophysics, Kyoto University, Kyoto, Japan; 5 Department of Atmospheric and Oceanic Sciences, University of California at Los Angeles, Los Angeles, USA; 6 Center for Nonlinear Plasma Science and C.R. ENEA Frascati, Frascati, Italy; 7 Institute of Fusion Theory and Simulation and School of Physics, Zhejiang University, Hangzhou, China; 8 Department of Physics and Astronomy, University of California, Irvine, USA

**Keywords:** Aurora, Magnetospheric physics

## Abstract

Chorus waves are naturally occurring electromagnetic emissions in space and are known to produce highly energetic electrons in the hazardous radiation belt. The characteristic feature of chorus is its fast frequency chirping, whose mechanism remains a long-standing problem. While many theories agree on its nonlinear nature, they differ on whether or how the background magnetic field inhomogeneity plays a key role. Here, using observations of chorus at Mars and Earth, we report direct evidence showing that the chorus chirping rate is consistently related to the background magnetic field inhomogeneity, despite orders of magnitude difference in a key parameter quantifying the inhomogeneity at the two planets. Our results show an extreme test of a recently proposed chorus generation model and confirm the connection between the chirping rate and magnetic field inhomogeneity, opening the door to controlled plasma wave excitation in the laboratory and space.

## Abstract (web summary)

Although whistler-mode chorus waves are common in the Earth’s and other planetary magnetospheres, the mechanism behind fast frequency chirping is debated. Here, the authors show the presence of chorus emissions at Mars, with fundamentally the same nonlinear nature as those at Earth, despite vastly different magnetic and plasma conditions.

## Introduction

Whistler-mode chorus waves are electromagnetic emissions frequently found in space and at other planets^1,2^. When the electromagnetic signal of chorus is converted to an acoustic signal, these waves sound like birds chirping at dawn, hence the name. Chorus waves are known to play a dominant role in accelerating relativistic electrons in Earth’s radiation belts^3,4^. They have also been demonstrated to scatter electrons with energies of a few hundred eV to a few keV into the atmosphere to form diffuse and pulsating auroras^5–7^, playing a crucial role in the energy and mass coupling between the ionosphere and magnetosphere^8^ and to be an embryonic source for plasmaspheric hiss^9,10^. Satellite observations show that chorus emissions are narrowband and quasi-coherent, consisting of discrete chirping elements with their central frequency changing rapidly as a function of time (i.e., chirping)^1,11^. The frequency chirping is not unique to whistler-mode chorus but has also been observed in electromagnetic ion cyclotron waves in space plasmas or Alfvén waves in fusion plasmas^12,13^. Hence there is a broad interest in understanding the fundamental physical mechanism of chorus chirping, in addition to the consensus on the importance of chorus. However, despite the early establishment of nonlinear wave–particle interactions in the generation of chorus, the theoretical mechanism of how nonlinear interactions lead to chorus chirping has been under intensive debate for over 70 years^14–18^.

A recently proposed “Trap-Release-Amplify” (TaRA) model^19^ shows that nonlinear wave–particle interactions and background magnetic field inhomogeneity work together to produce the chirping of chorus in space, unifying two different ways of estimating chorus chirping rate from previous theoretical models^14,16^. As a self-consistent model, electrons interact with the quasi-coherent wave packet, resulting in quasi-coherent rather than the stochastic motion of a small group of resonant electrons. The quasi-coherent motion leads to significant changes in the electron’s energy and momentum. Because the phase space density along an electron’s trajectory is constant, there is a difference between the electron’s phase space density and its neighboring at the new location, leading to the formation of phase-space structures such as phase space holes. This nonlinear interaction process results in a frequency chirping rate proportional to the wave amplitude at the equator, a conclusion verified for chorus events at Earth^20,21^ and is widely accepted^22,23^. The main unique feature of the TaRA model is that it further predicts that the chirping rate is proportional to the local magnetic field inhomogeneity when the quasi-coherent phase-space structure is released from the wave packet and leads to selective amplification of new narrowband emissions. This prediction of the TaRA model is consistent with one of the previous theoretical models of chorus^14^ and the statistical dependence of the chirping rate of chorus on the radial distance at Earth^24^. However, different from the nonlinear chirping rate, which is a defining feature of chorus, the dependence of the chirping rate on the magnetic field inhomogeneity is still under debate^22^. Note that the change of inhomogeneity of the background magnetic field at Earth is relatively mild. A direct experimental test with orders of magnitude variation in magnetic field inhomogeneity is not possible using wave observations only at Earth.

In this work, we conduct a detailed analysis of a previously reported chorus-like emission observed by the Mars Atmosphere and Volatile EvolutioN (MAVEN) mission^25^. Despite the vastly different magnetic field and plasma parameters, we demonstrate that the emission observed by MAVEN at Mars shares the same nonlinear nature with chorus waves at Earth and exhibits features consistent with the predictions made by the TaRA model.

## Results

### Observation

**Fig. 1:**
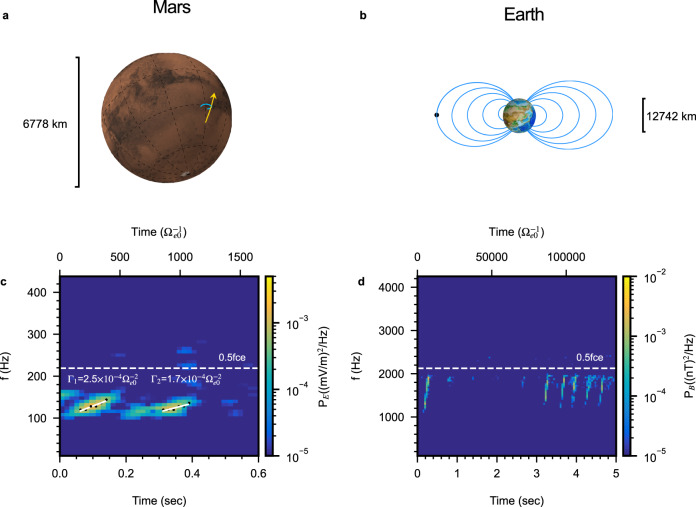
Magnetic field and chorus emissions at Mars and Earth. Traced magnetic field lines, represented by blue lines, at Mars (**a**) and Earth (**b**) are shown. The scale bars indicate the size of each planet. The yellow arrow in panel (**a**) denotes the trajectory of MAVEN, and the black dot in panel (**b**) indicates where chorus waves shown in panel (**d**) were observed. Frequency–time spectrograms of chorus waves observed at Mars (**c**) by MAVEN and Earth (**d**) by Van Allen Probe B are shown. Color-coded is the power spectrum density calculated using the wave electric field (**c**) and magnetic field (**d**). The time coordinate indicates the number of seconds since 2015-07-12/06:00:53 UT for panel (**c**) and 2012-10-08/06:03:57 UT for panel (**d**). The white dashed lines in (**c**) and (**d**) indicate half electron cyclotron frequency. Black dots in panel (**c**) denote the maximum power spectral density at a given time, and the white line represents the linear least-squares fitting, whose slope gives the frequency chirping rate shown in normalized units.

MAVEN detected the wave event on 12 July 2015, and linear instability analysis confirms that the wave is of whistler mode^26^. However, linear analysis can neither confirm this event as chorus nor explain its frequency chirping, which is fundamentally nonlinear. Figure 1a shows the trajectory of the MAVEN satellite and the magnetic field line it crosses while observing the event, whose frequency–time spectrogram is shown in Fig. 1c. Note that Mars does not have a global magnetic field like Earth. The closed magnetic field line is part of the crustal magnetic field of Mars. As a comparison, we also show in Fig. 1b the global magnetic field and a chorus event of Earth observed by the Van Allen Probes mission^27^ (Fig. 1d). The crustal magnetic field line at Mars encountered by MAVEN has a much smaller scale than that of typical magnetic field lines at Earth. The inhomogeneity parameter (*ξ*) of a given magnetic field line, relevant to studies of chorus, can be obtained by approximating the magnetic field strength near the equator (minimum *B*) by a parabolic function; i.e., *B* = *B*_0_(1 + *ξs*^2^), where *s* is the distance from the equator along a field line. Using corresponding magnetic field models, we obtain that, for the chorus event at Earth, *ξ*_E_ ≈ 5 × 10^−9^ km^−2^, and for the event at Mars, *ξ*_M_ ≈ 1.4 × 10^−4^ km^−2^. The five orders of magnitude difference between the inhomogeneity factor *ξ* provide a unique opportunity for an extreme test of the dependence of chorus properties on background magnetic field inhomogeneity.

### Nonlinear chirping rate

**Fig. 2:**
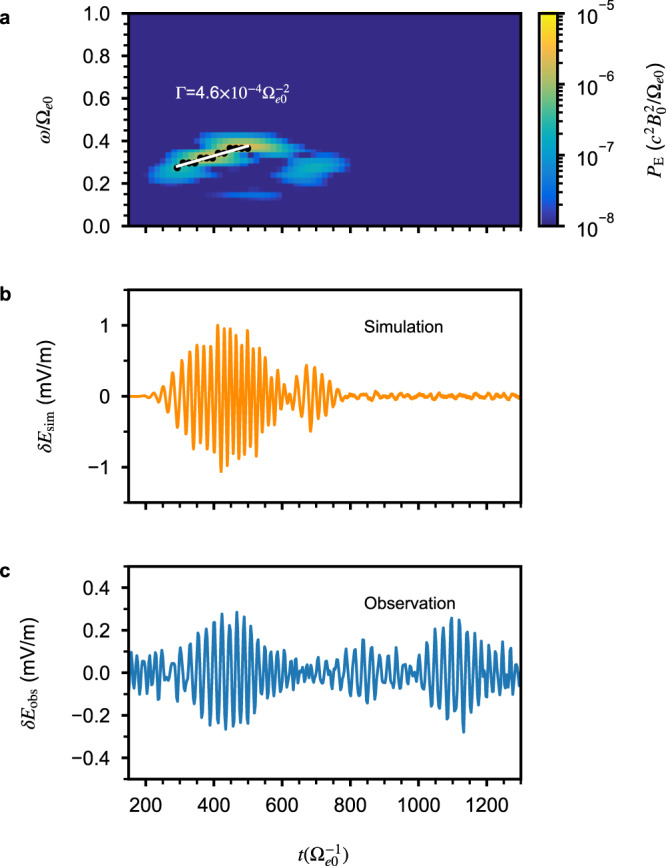
Computer simulation of the chorus event at Mars. **a** The simulated rising-tone chorus waves obtained using the electric fields at *s*/*d*_*e*_ = 0.38, corresponding to the position of the observed chorus event. Color-coded is the power spectral density of the wave electric field in normalized units. The black dots and white line are the same as those in Fig. 1c. **b**, **c** Comparison of waveform from simulation and observation.

To prove that the waves presented in Fig. 1c at Mars are indeed chorus emissions, we need to demonstrate that the frequency chirping is due to nonlinear wave–particle interactions. Because of the instrument resolution limitations, directly identifying nonlinear phase-space structures in the observed electron distribution is not possible. Therefore, we perform a self-consistent computer simulation using the observed particle distribution and the local magnetic field line model (see “Methods”, subsection “Computer simulation setup”). Figure 2a shows the simulated wave event, which exhibits the same chirping characteristics as the observed event in Fig. 1c. The chirping rate of the element from the computer simulation is $4.6\times 1{0}^{-4}{\Omega }_{e0}^{-2}$, whereas the chirping rates of the two observed elements are 2.5 × 10^−4^ ${\Omega }_{e0}^{-2}$ (or 306 Hz/s) and 1.7 × 10^−4^ ${\Omega }_{e0}^{-2}$ (or 205 Hz/s). Here Ω_*e*0_ ≡ *eB*_0_/*m* is the electron angular cyclotron frequency at the equator, with *e* the elementary charge and *m* electron mass. Therefore, the chirping rate from simulation and observation differs by about a factor of two to three. Figure 2b shows the wave electric field from simulation and observation in physical units. The electric field amplitude from the simulation is consistent with that from observation within a factor of three. Note that the computer simulation does not reproduce the second element from observation, which is expected and commonly seen in other simulations of chorus, because no free energy is re-supplied to the simulation system after the generation of the first element^28,29^. The slight difference in chirping rate between the first and second elements may indicate a change in electron distribution, as noted in previous studies^30,31^. However, due to the limited time resolution (2 s) of the SWEA instrument onboard MAVEN, this difference cannot be resolved. Overall, the simulation results exhibit good consistency with the observed data for both the chirping rate and wave amplitude, despite uncertainties in the simulation parameters. Therefore, the successful reproduction of the observed event by computer simulation enables us to obtain parameters that are not possibly available from direct observation.

Now we confirm that the frequency chirping observed by MAVEN and from the computer simulation is caused by a nonlinear process, a characteristic feature of chorus waves. Nonlinear wave–particle interaction theories^16–19^ predict a chirping rate (Γ_NL_), which is proportional to the wave magnetic amplitude (*δB*) at the equator. This chirping rate is also proportional to a parameter *R*, which characterizes the nonlinear wave–particle interactions and is typically within the range of 0.2 ~ 0.8 (see “Methods”, subsection “Theoretical estimate of the chirping rate”). Note that the event from observation is off the equator; therefore, the theoretical chirping rate cannot be directly calculated using the measured wave amplitude. Nevertheless, we may test if the nonlinear chirping rate is of the same order as that from observation. Using the average electric field from observation and the whistler wave dispersion relation, the wave magnetic field amplitude is estimated to be approximately *δB*/*B*_0_ ≈ 10^−2^. Using plasma parameters determined from MAVEN observation, we estimate that for *ω* = 0.3Ω_*e*0_, Γ_NL_ ≈ 4.5 × 10^−4^ ${\Omega }_{e0}^{-2}$ and 1.8 × 10^−3^ ${\Omega }_{e0}^{-2}$ with *R* = 0.2 and 0.8, respectively. Correspondingly, considering the crude nature of the estimate and that the event is off the equator, the theoretical nonlinear chirping rate is in the same order as the observed event.

**Fig. 3:**
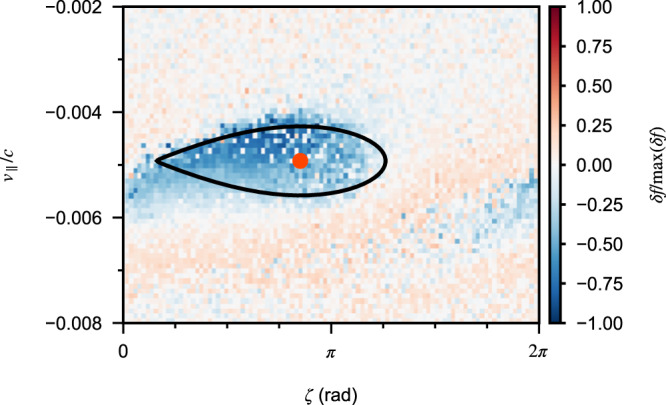
Electron phase-space structure. The *v*_∥_-*ζ* phase-space distribution at *s* = 0 and *v*_⊥_ = 0.0085*c*, with *ζ* the angle between particle perpendicular velocity and wave magnetic field, from the computer simulation of the chorus event at Mars. The phase-space hole is clearly seen with the center near *v*_∥_ = −0.005*c*. A fitting to the boundary of this hole is performed using the motion of resonant electrons with *R* = 0.45. The black line indicates the separatrix and the red dot marks the corresponding center.

A more accurate determination of the nonlinear nature of the chirping is via the phase-space dynamics of electrons from simulation. Incidentally, this can also verify the nonlinear chirping rate, because the parameter *R* is defined by the chirping rate and can be estimated directly from the electron phase-space structure from the computer simulation of the event^16,17,32^. Figure 3 shows the electron phase-space distribution at the equator near the resonance velocity, which clearly confirms the presence of the characteristic phase-space hole associated with the generation of a rising-tone chorus. By performing a rough fitting to the boundary of the phase-space hole, we obtain directly that *R* ≈ 0.45. Correspondingly, the nonlinear chirping rate is naturally valid with this value of *R* at the equator. The analysis of the electron phase-space dynamics confirms the nonlinear chirping rate predicted by several models^15–18^. More importantly, it demonstrates that the wave event observed by MAVEN is generated by a nonlinear process, similar to chorus emissions observed at Earth.

### Chirping rate from magnetic field inhomogeneity

A unique feature of the TaRA model is that, besides the nonlinear chirping rate at the equator, it also predicts that the chirping rate is related to the magnetic field inhomogeneity at the source location, denoted by *s*_0_. Physically this is because of the phase-locking condition, which requires the balance between the wave chirping, characterized by a term *R*_1_, and the background magnetic field inhomogeneity, characterized by a term *R*_2_. The result of this balance is a theoretical chirping rate, Γ_IN_, that is proportional to the magnetic field inhomogeneity at the source location *s*_0_ where the wave amplitude is negligibly small (see “Methods”, subsection “Theoretical estimate of the chirping rate”). The location *s*_0_ could be roughly given by the interaction region size based on the linear motion of electrons or more accurately estimated directly from simulation. Again, we first use a Mars crustal magnetic field model to test if using the magnetic field inhomogeneity gives the correct order of magnitude estimate of the chirping rate. For this rough estimate, we use ${s}_{0}={(2\pi {v}_{r}/\xi {\Omega }_{e0})}^{1/3}$, which corresponds to a shift of *π* radian in wave–particle interaction phase angle from the equator by assuming that electrons move adiabatically along the background magnetic field^14^. Here *v*_*r*_ is the electron resonance velocity. For the chorus event at Mars, this estimate gives *s*_0_ ≈ −0.28*d*_*e*_ with *d*_*e*_ ≡ *c*/Ω_*e*0_, and the theoretical chirping rate Γ_IN_ is about $2\times 1{0}^{-3}{\Omega }_{e0}^{2}$. This value is comparable to the estimate of the nonlinear chirping rate with *R* = 0.8 and larger than the chirping rate from the simulation by about a factor of four or the observed one by a factor of ten. As shown below, the discrepancy between the theoretical and the observed or simulated chirping rate for the case of Mars is mainly due to the rough nature of *s*_0_ estimated from the linear motion of electrons.

**Fig. 4:**
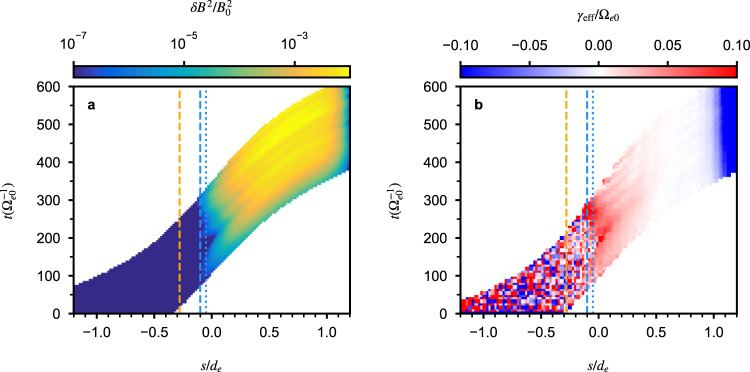
Wave propagation and effective growth rate. **a** The wave magnetic field strength and **b** the effective growth rate as a function of *s* and *t* for the wave propagating in the *s* direction, from the computer simulation of the chorus event at Mars. The orange dashed lines mark *s*/*d*_*e*_ = −0.28, while the blue dashed and dotted lines mark *s*/*d*_*e*_ = −0.1 and *s*/*d*_*e*_ = −0.05, respectively.

For a more direct comparison of the Mars event, we focus on the simulated event and use the information from computer simulation to find a better estimate of the source location *s*_0_. Figure 4 shows the wave propagation and the effective growth rate for the event of interest from simulation. The source location *s*_0_ could be estimated from the figure of the effective growth rate *γ*_eff_ as the location between smoothly varying regions and noisy-like regions due to background thermal noise. Clearly, for this particular case, the above crude estimate of *s*_0_ based on the linear motion of electrons is too large; it should be roughly between −0.05*d*_*e*_ and −0.1*d*_*e*_ for the simulated case.

**Fig. 5:**
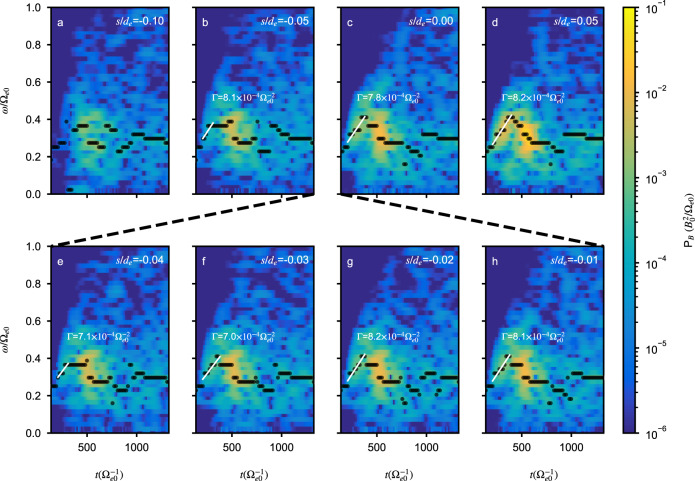
Wave magnetic field spectrogram upstream from the equator. Black dots mark the maximum wave power spectral density for a given time for the simulated chorus event at Mars. White lines denote a linear least-squares fitting to the corresponding black dots starting from the fifth black dot. The fitting for the spectrograms at *s*/*d*_*e*_ = −0.04 and −0.05 uses seven data points in total due to their weak intensity. In general, the chirping rate estimate is more reliable for chorus elements with a stronger wave signal.

Figure 5 displays wave spectrograms for the interval *s*/*d*_*e*_ = −0.1 to *s*/*d*_*e*_ = 0.05 to support the estimated value of *s*_0_ discussed above. At *s*/*d*_*e*_ = 0 (Fig. 5c) and 0.05 (Fig. 5d), clear chirping elements are observed around *t*Ω_*e*0_ = 300, characterized by chirping rates of $7.8\times 1{0}^{-4}{\Omega }_{e0}^{-2}$ and $8.2\times 1{0}^{-4}{\Omega }_{e0}^{-2}$, respectively. The chirping rate at *s*/*d*_*e*_ = 0.38 differs from that at *s*/*d*_*e*_ = 0 due to wave packet distortion during propagation. It should be noted that the stronger signal near *t*Ω_*e*0_ = 500 in these spectrograms should not be interpreted as a falling tone in Fig. 5c–d or bi-directional chirping elements in other panels. No corresponding nonlinear phase-space structure can be found in the electron distribution, and these spectral structures do not maintain a consistent shape. At *s*/*d*_*e*_ = −0.05, a weak chirping structure around *t*Ω_*e*0_ = 300 is visible. To establish a connection between this structure and the chorus element at the equator, we present wave spectrograms at intermediate locations between *s*/*d*_*e*_ = −0.05 and 0 in Fig. 5e–h. These spectrograms clearly illustrate the amplification of the chirping element as it propagates from *s*/*d*_*e*_ = −0.05 to *s*/*d*_*e*_ = 0, supporting that the source region lies upstream of −0.05. Furthermore, the chirping elements exhibit consistent chirping rates; hence, we will use the chirping rate at the equator for the following analysis. On the other hand, at *s*/*d*_*e*_ = −0.1 (Fig. 5a), the wave signal is comparable to background noise between *t*Ω_*e*0_ = 200 and 300. Taken together, these spectrograms show the chorus element’s source location is between *s*/*d*_*e*_ = −0.05 and −0.1.

**Fig. 6:**
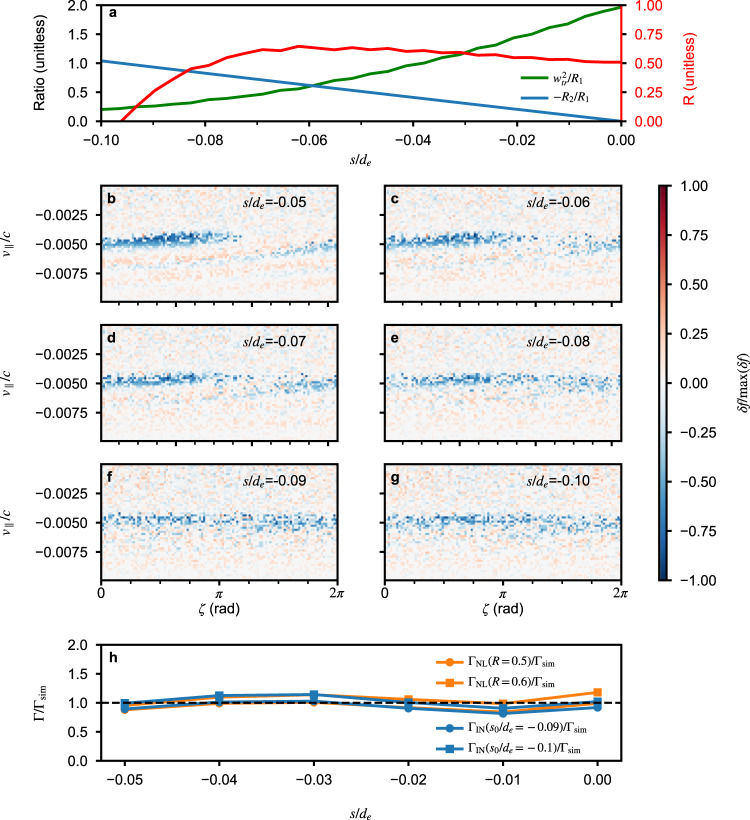
Electron phase-space dynamics and comparison of chirping rates upstream from the equator. **a** Variation of ${\omega }_{tr}^{2}/{R}_{1},-{R}_{2}/{R}_{1}$, and *R* as a function of *s*. Panels **b**–**g** show the electron phase-space (*v*_∥_-*ζ*) distribution between *s*/*d*_*e*_ = −0.05 and −0.1. Panel **h** displays the ratio of theoretical chirping rates to the chirping rate from simulation between *s*/*d*_*e*_ = −0.05 and 0 for the wave event at Mars. The nonlinear chirping rate is estimated using the general definition of *R* for off-equatorial locations, with *R* = 0.5 and 0.6. The chirping rate from inhomogeneity is estimated using *s*_0_/*d*_*e*_ = −0.09 and −0.1.

To further constrain the value of *s*_0_ and verify the balance of *R*_1_ and *R*_2_ or the theoretical chirping rate Γ_IN_, we analyze the electron phase-space dynamics and compare the chirping rate in Fig. 6. In Fig. 6a, we plot the ratio of a nonlinearity term ${\omega }_{tr}^{2}$ (see “Methods”, subsection “Theoretical estimate of the chirping rate”) and the inhomogeneity term *R*_2_ to the wave chirping term *R*_1_ as a function of *s*/*d*_*e*_ between −0.1 and 0, together with the parameter *R*. The wave amplitude is estimated from the square root of the integrated wave power spectral density. As can be seen from the plot, the ratio ${\omega }_{tr}^{2}/{R}_{1}$ decreases from approximately 2 at *s* = 0 to around 0.2 at *s*/*d*_*e*_ ~ −0.1. Note that due to simulation noise, this term can never reach zero in a particle simulation. On the other hand, the ratio −*R*_2_/*R*_1_ increases rapidly from 0 at *s*/*d*_*e*_ = 0 to about 1.04 at −0.1 due to the extremely large inhomogeneity factor of the background magnetic field. The parameter *R* varies between 0.5 and 0.6 between *s*/*d*_*e*_ = −0.07 and 0 and decreases to 0 at approximately *s*/*d*_*e*_ = −0.096, where *R*_1_ = −*R*_2_. To support the estimated value of *R* in Fig. 6a, we display electron phase-space distributions between *s*/*d*_*e*_ = −0.05 and −0.1 in Fig. 6b–g, which exhibit good consistency with the variation of *R*. These analyses suggest that the source location *s*_0_ lies within the range of −0.1 ≲ *s*_0_/*d*_*e*_ ≲ −0.09. Using this information about *s*_0_, we show the ratio of the chirping rates from nonlinear theory (Γ_NL_) and inhomogeneity (Γ_IN_) to those from simulation in Fig. 6h. The comparison suggests that the chirping rate from inhomogeneity agrees with the rates obtained from both simulation and the well-established nonlinear theory.

## Discussion

The typical inhomogeneity factor of the global magnetic field of Earth is about five orders of magnitude smaller than that of the crustal magnetic field of Mars used by this study. However, a previous analysis involving 1106 chorus elements has shown that the theoretical chirping rate from inhomogeneity exhibits good statistical agreement with the observed chorus elements at Earth^24^. For the Earth chorus event shown in Fig. 1d, a theoretical estimate, with observed plasma parameters, gives that the observed chirping rate (Γ_obs_) is approximately twice the theoretical rate obtained from inhomogeneity (Γ_IN_) (see “Methods”, subsection “The frequency chirping rate calculation of Earth chorus waves”). These previous statistical results at Earth and the current result at Mars demonstrate that the chorus chirping rate is consistently related to the background magnetic field inhomogeneity, despite a few orders of magnitude difference in the inhomogeneity factor. Therefore, the combined results of these studies suggest that the physics involved in obtaining the chirping rate from inhomogeneity is as important as those involved in nonlinear wave–particle interactions for chorus emissions at these planets.

The above comparison between observation, simulation, and theory proves the presence of chorus emissions at Mars, with fundamentally the same nonlinear nature as those at Earth. Furthermore, it presents an extreme test of one of the TaRA model predictions that the chirping rate is related to the background magnetic field inhomogeneity besides being proportional to wave amplitude at the equator. It demonstrates that the chirping of chorus results from a complex interplay between nonlinear wave–particle interactions and the magnetic field inhomogeneity. The consistency in the two kinds of chirping rate between observation and theory further establishes the validity of the recently proposed TaRA model for the event. Future studies involving a large number of chorus events are needed to statistically test the TaRA model. The relation between the chirping rate and magnetic field inhomogeneity may be used to infer the background magnetic field inhomogeneity of Mars or any other planets from chorus observation, while the nonlinear chirping rate can be used to estimate the chorus wave amplitude. These results allow a more controlled setup in future active experiments of plasma waves or radiation belt remediation through chorus emissions.

## Methods

### Computer simulation setup

The study employs the self-consistent particle simulation code DAWN^33^ to simulate a system along the magnetic field line, considering only parallel-propagating waves. The simulation treats cold electrons as a fluid and models hot electrons using the nonlinear *δf* method^34^ to minimize simulation noise. Particle boundaries are set as reflecting, while wave boundaries are absorbing. A grid size of 6 × 10^−4^ *d*_*e*_ is used to ensure the proper resolution of wavelength, which is approximately 0.04 *d*_*e*_ at the equator for the emission observed by MAVEN. A time step of $3\times 1{0}^{-4},{\Omega }_{e0}^{-1}$ is used to satisfy the Courant condition. The simulation uses 4000 cells and 2000 simulation particles per cell for each electron population.

**Fig. 7:**
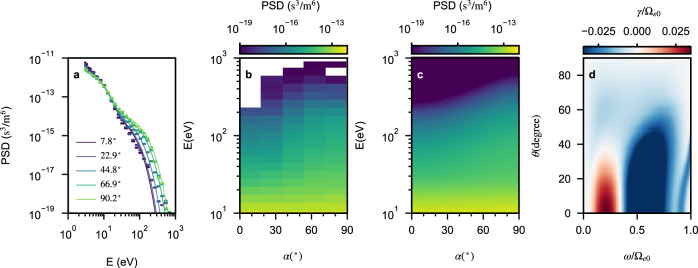
Electron distribution and linear growth rate. **a** Comparison of measured and fitted electron PSD as a function of energy at different pitch angles. Dots represent measured values, while lines show fitted results. **b** Two-dimensional electron PSD as a function of pitch angle (*α*) and energy from MAVEN measurements. **c** Two-dimensional electron PSD from fitting. **d** Two-dimensional linear growth rate calculation, showing that the linear growth rate peaks for parallel-propagating waves with frequencies near 0.21 ~ 0.23Ω_*e*0_. According to chorus wave excitation theories^17,19^, the frequency of the maximum linear growth rate roughly corresponds to the starting frequency of the chorus element. Correspondingly, the linear growth rate calculation is consistent with observation.

To improve computational efficiency while representing the observed electron distribution by MAVEN, our simulation employs two populations of hot electrons. Figure 7a shows the electron phase-space densities (PSDs) as a function of energy obtained from the SWEA measurements^35^ at 2015-07-12/06:00:53 UT. Linear instability analysis shows that the growth rate dominates in the parallel direction. We obtained the equatorial distributions of hot electrons by fitting the electron velocity distribution function with the sum of two bi-Maxwellian functions. The function form is given by

1 $$f\left({u}_{\parallel },{u}_{\perp }\right)=\frac{1}{{(2\pi )}^{3/2}{w}_{\parallel }{w}_{\perp }^{2}}\exp \left(-\frac{{u}_{\parallel }^{2}}{2{w}_{\parallel }^{2}}-\frac{{u}_{\perp }^{2}}{2{w}_{\perp }^{2}}\right),$$

in which *u*_∥_ and *u*_⊥_ are velocities parallel and perpendicular to the background magnetic field, and *w*_∥_ and *w*_⊥_ are corresponding thermal velocities in the non-relativistic limit. Fitting results reveal that the first component has a plasma frequency of 48.6Ω_*e*0_ and thermal temperatures of *T*_∥_ = 2.9 eV and *T*_⊥_ = 4 eV, while the second component has a plasma frequency of *ω*_*pe*_ = 168.5Ω_*e*0_ and thermal temperatures of *T*_∥_ = 20 eV and *T*_⊥_ = 41 eV. We set the plasma frequency of the cold electrons to 128Ω_*e*0_ to ensure the total electron number density is consistent with the observed value (*n*_*e*_ = 112 cm^−3^).

### Theoretical estimate of the chirping rate

The TaRA model^19^ estimates the chirping rate based on the following equation for the wave–particle interaction phase angle *ζ* ≡ 〈***v***_⊥_, *δ****B***〉,

2 $$\frac{{{{{{{{{\rm{d}}}}}}}}}^{2}\zeta }{{{{{{{{\rm{d}}}}}}}}{t}^{2}}={\omega }_{tr}^{2}\sin \zeta -({R}_{1}+{R}_{2}).$$

Here ${\omega }_{tr}^{2}\equiv k{v}_{\perp }e\delta B/m$ is the phase trapping frequency squared, with *k* the wave number and *v*_⊥_ the component of the resonant electron velocity perpendicular to the local magnetic field. The two parameters *R*_1_ and *R*_2_ are defined by

3 $${R}_{1}={\left(1-\frac{{v}_{r}}{{v}_{g}}\right)}^{2}\frac{\partial \omega }{\partial t},$$

4 $${R}_{2}=\left(\frac{k{v}_{\perp }^{2}}{2{\Omega }_{e}}-\frac{3{v}_{r}}{2}\right)\frac{\partial {\Omega }_{e}}{\partial s},$$

where *v*_*g*_ is the wave group velocity, and Ω_*e*_ is the electron cyclotron frequency at *s*. The parameter *R*, which characterizes the nature of electron dynamics, is defined by $R\equiv ({R}_{1}+{R}_{2})/{\omega }_{tr}^{2}$. At the equator, where the background magnetic field inhomogeneity is negligible (*R*_2_ → 0), the nonlinear chirping rate can be obtained from the definition of *R*, i.e.,

5 $${\Gamma }_{{{{{{{{\rm{NL}}}}}}}}}={\left.R{\left(1-\frac{{v}_{r}}{{v}_{g}}\right)}^{-2}{\omega }_{tr}^{2}\right|}_{s=0}.$$

Based on previous theories on nonlinear wave–particle interactions^16–18^, the value of *R* typically falls between 0.2 and 0.8 to maximize wave–particle power transfer. On the other hand, at the wave source location (*s*_0_) upstream, the amplitude of the wave is comparable to that of the background noise and ${\omega }_{tr}^{2}\ll {R}_{2}$. By the principle of selective amplification, the TaRA model requires that the phase-locking condition (d^2^*ζ*/d*t*^2^ = 0) is satisfied at *s*_0_. Correspondingly, we obtain a chirping rate proportional to the magnetic field inhomogeneity at *s*_0_, i.e.,

6 $${R}_{1}\approx -{R}_{2}\Rightarrow {\Gamma }_{{{{{{{{\rm{IN}}}}}}}}}\approx -{\left(1-\frac{{v}_{r}}{{v}_{g}}\right)}^{-2}\left(\frac{k{v}_{\perp }^{2}}{2{\Omega }_{e}}-\frac{3{v}_{r}}{2}\right){\left.\frac{\partial {\Omega }_{e}}{\partial s}\right|}_{s={s}_{0}}.$$

Therefore, the TaRA model recovers the chirping rate originally proposed by Helliwell^14^, besides the nonlinear chirping rate. When estimating the two theoretical chirping rates Γ_NL_ and Γ_IN_, the perpendicular velocity *v*_⊥_ of the resonant particle is obtained with a pitch angle of 70° from nonlinear wave–particle interaction theories and the wave number *k* is determined by the whistler wave dispersion relation.

### Determining the background magnetic field inhomogeneity at Mars

**Fig. 8:**
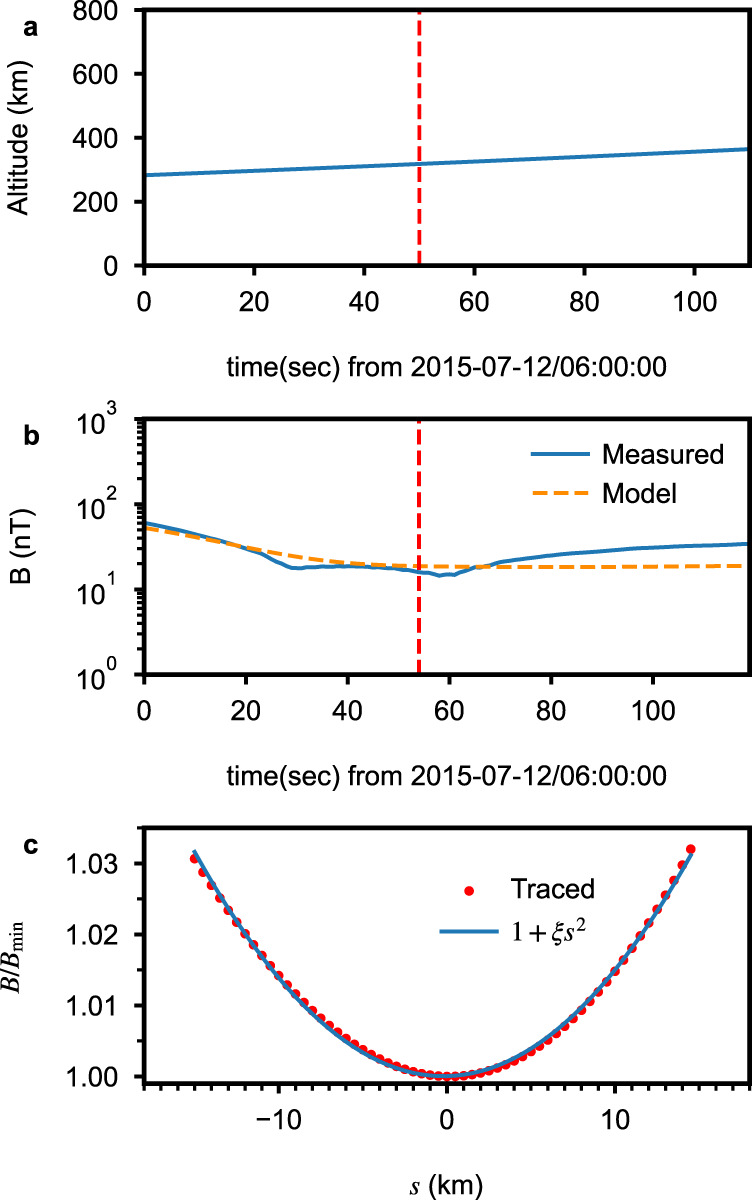
MAVEN altitude and magnetic field from observation and model. **a** The altitude of MAVEN is shown, while **b** compares the magnetic field strength from MAVEN observations to the Morschhauser crustal magnetic field model. The vertical dashed line indicates the time of the analyzed chorus event. **c** The traced (dots) and fitted (solid line) magnetic field strengths near the equator (minimum B) are compared, with the inhomogeneity factor *ξ* determined from the coefficient of the parabolic fitting.

Figure 8a shows that MAVEN was at an altitude of approximately 300 km during the chorus event observation. The magnetic field measured by MAVEN’s MAG instrument matches well with the widely used Morschhauser crustal magnetic field model^36^, as shown in Fig. 8b. This indicates that the crustal magnetic field of Mars dominates during the event of interest. To determine the inhomogeneity factor *ξ* at Mars, we use the Morschhauser magnetic field model and trace the magnetic field line in both directions from the observing location (altitude: 325 km, longitude: 211°, and latitude: −31. 8°). At the time of observation (about 06:00:54 UT), the tracing result reveals that the observation point is not too far away from the magnetic field minimum (B${}_{\min }$). We fit the magnetic field magnitude near the magnetic field minimum as a function of distance, shown in Fig. 8c. For this fitting, we conclude that the normalized background magnetic field inhomogeneity parameter is $\xi=1.4\,{d}_{e}^{-2}$.

### The frequency chirping rate calculation of Earth chorus waves

For the chorus event observed at Earth shown in Fig. 1d, the observed frequency chirping rate is about 6000 Hz/s. The measured background magnetic field is 152 nT, and the electron density is 2 cm^−3^. The inhomogeneity of the background magnetic field is estimated to be 5.45 × 10^−9 ^km^−2^ using T89 magnetic field model^37^. With wave number *k* determined from the whistler wave dispersion relation for *f* = 0.35*f*_*ce*_ ≈ 1500 Hz and *v*_⊥_ determined from the resonance velocity with a pitch angle of 70°, the chirping rate Γ_IN_ is calculated to be approximately 3529 Hz/s, agrees with the observed one within a factor of two.

## Data Availability

The datasets generated during the current study have been deposited in a Zenodo repository and are openly available at 10.5281/zenodo.7844698^38^. MAVEN wave data are publicly available through https://lasp.colorado.edu/maven/sdc/public/data/sci/lpw/l2/. MAVEN particle data are publicly available through https://lasp.colorado.edu/maven/sdc/public/data/sci/swe/l2/. MAVEN magnetic field data are publicly available through https://lasp.colorado.edu/maven/sdc/public/data/sci/mag/l2/. Van Allen Probes wave data are obtained from https://emfisis.physics.uiowa.edu/Flight/. Source data are provided with this paper.

## Source data

Source Data

## Code availability

The DAWN simulation code is available from the corresponding author upon request. The GEOPACK package for the Earth’s magnetic field is publicly accessible from https://pypi.org/project/geopack/. The MAVEN toolkit used to calculate the magnetic field of Mars is publicly accessible from http://lasp.colorado.edu/maven/sdc/public/data/sdc/software/idl_toolkit/Toolkit_V2019-09-25_Public.zip.
